# Indonesian adolescents’ perspectives on smoking habits: a qualitative study

**DOI:** 10.1186/s12889-020-10090-z

**Published:** 2021-01-07

**Authors:** Fithria Fithria, Muhammad Adlim, Syarifah Rauzatul Jannah, Teuku Tahlil

**Affiliations:** 1 Graduate School of Mathematics and Applied Sciences, Universitas Syiah Kuala, Banda Aceh, 23111 Indonesia; 2 Department of Family Health Nursing, Faculty of Nursing, Universitas Syiah Kuala, Banda Aceh, 23111 Indonesia; 3 Department of Psychiatry and Mental Health Nursing, Faculty of Nursing, Universitas Syiah Kuala, Banda Aceh, 23111 Indonesia; 4 Department of Community Health Nursing, Faculty of Nursing, Universitas Syiah Kuala, Banda Aceh, 23111 Indonesia

**Keywords:** Smoking habit, Islamic perspectives, Adolescent, Smoking prevention, Indonesia

## Abstract

**Background:**

The prevalence of smoking among adolescents is high in Indonesia. Therefore, this qualitative research aimed to explore the perspectives of Muslim adolescents on smoking habits as a reference for developing effective prevention programs.

**Methods:**

Three focus group discussions involving 24 junior high school male students (mean age = 13.75 years) were the main source of data for this phenomenological qualitative study. The discussion guide was developed by the researchers based on the reviewed literature and validated by experts. The research findings were analyzed using an inductive content analysis with systematic steps based on the stages of qualitative data analysis.

**Results:**

Adolescent perspectives on smoking were grouped into two themes: perception of smoking and smoking-related factors. The perception of smoking encompassed three sub-themes: smoking as a social habit, contradictive feelings, and the Islamic perspective. The smoking-related factors included peer pressure, the parents’ smoking status, masculinity and curiosity. The results indicated that adolescents consider smoking as a social habit but with contradictory feelings. The smoking habit was also stimulated by peer pressure, imitating parents who smoke, feeling masculine and curiosity.

**Conclusion:**

We suggest that health professionals who are interested in developing smoking prevention programs in Indonesia should consider the adolescent perspective on smoking so that the prevention program will be more effective and appropriate for adolescents.

## Background

Smoking causes social, economic and health problems [1–3]. Various studies have shown that smoking is a risk factor for cardiovascular diseases, stroke, and various types of cancer [4, 5]. However, the percentage of smokers is still high in Indonesia. According to data from 2013, Indonesia had the third highest prevalence of smoking among the nine countries in North and Southeast Asia [6]. In addition, a previous study indicated the smoking prevalence in Indonesia did not decline significantly between 2007 and 2014 [7]. Among the 30 countries with a high Muslim population, Indonesia is ranked the second highest for prevalence of smokers [8]. A previous study in Indonesia showed that smoking is more prevalent among men than women [6].

Smoking is also high among Indonesian adolescents; a national survey in 2006 showed that of 3737 students aged 13 to 15 years, 37.7% had smoked cigarettes, 13.5% were identified as current tobacco smokers, 11.8% were current cigarette smokers, and 3.8% used other tobacco products. Furthermore, 95.1% of adolescents who stated that they had never tried any tobacco wanted to use it in the next 12 months [9]. In 2014, the Global Youth Tobacco Survey (GYTS) in Indonesia showed that 20.3% of 13- to 15-year-old students were using any tobacco products, 19.4% were smoking tobacco, 18.3% were smoking cigarettes, and 2.1% were using smokeless tobacco [10].

This shows a tendency for increasing numbers of smokers. Hence, an effective prevention effort must be prioritized because it has been shown that adolescents who tried smoking at the age of 10–14 years were predicted to continue for the next 2 years, and smoking behavior during adolescence is a predictor of smoking status in the future [11]. These statistics require serious attention because adolescents play an important role as a human resource of the future.

Many factors contribute to smoking among adolescents. Newly enrolled high school students aged 12–13 years are at high risk of smoking; they start to believe that smoking could reduce their apprehension about the regulations and social interactions in the school [12]. Previous studies have indicated that the highest rates of smoking among adolescents occurred during the transition to high school due to psychologic distress [13]. The students believe that smoking helps them to adapt to the physical, cognitive, and emotional changes that are taking place, although various studies have proved that smoking reduces self-esteem and self-image among those with severe addiction [14]. A previous study also showed that smoking addiction is associated with depression; but adolescents perceived that smoking could eliminate their negative feelings, and this perception was one of the risk factors for failure in a cessation program [15]. A previous report also found that teenagers are vulnerable to negative social influences from commercial groups that promote cigarettes, and therefore building their self-confidence is crucial to reduce vulnerability [4].

Another smoking vulnerability for adolescents comes from their siblings and friends [16]. In some communities, the smoker usually has emotional connections with his/her friends so that smoking becomes a socio-cultural identity and the smoking habit is accepted as a normative practice. Similar perceptions and behaviors will be adopted by the youth who are part of this community and interact with those groups [17]. An earlier report also found that youth smokers were usually in low socio-economic circles, from broken families with addicted parents [5] and household conflict [18].

In addition to these factors, religion also influences smoking habits. In the United States, increased doctrinism was associated with reduced risks [19]. In a Turkish study, fewer religious individuals smoked compared with non-religious individuals [20]. A study conducted in China also reported that pious Muslim men were mostly non-smokers [21]. Therefore, religious approaches can be effective in programs for cigarette and drug dependency, and Islamic teachings have been reported to be effective in preventing children from starting this behavior [22].

This qualitative research provides a specific description of the phenomenon of smoking among Indonesian Muslim adolescents, which focuses on adolescents’ perspective as a whole, including the Islamic viewpoint and other related factors.

## Methods

### Study design and participants

This is a phenomenological study, which used focus group discussions (FGDs) to explore the perspectives of Muslim adolescents on smoking. For the qualitative phase of the study [23], students from three junior high schools representing three sub-districts in Aceh Besar were chosen as the participants. These schools are suburban, with good public transportation and located less than 20 km from the capital of Aceh Province. Based on the district statistics in 2017, the total population in the district was 409,109 inhabitants (around 2000 people/km^2^), with an average household size of 4, a poverty level of 15.41%, labor force participation rate of 59.17%, and open unemployment rate of 8.49%; service, trading, and agriculture are the main sources of income [24]. The three schools were chosen based on the following inclusion criteria: located in Aceh Besar district, with the same accreditation level (B), and in a community with moderate life conditions.

Participants were chosen by purposeful selection according to the following inclusion criteria: male students, aged 12 to 18 years, living with parents, and able to communicate in the Indonesian language. The exclusion criteria were students less than 12 years old or more than 18 years old and unable to communicate well in the Indonesian language. The researchers asked permission from the school management before approaching the students. Then, the students were informed about the aim of the study and the study procedure. The researcher approached the participants and their parents by phone before collecting data.

Written consent to participate in this study was obtained from participants’ parents, except one participant who was 18 years old gave his consent directly. The written consent form was sent to parents via the school management. Twenty-four participants, eight students from each school, were eligible to take part in the research. All communications with the students and their parents were conducted via their school management.

### Data collection and procedure

The researchers attempted to build relationships with the participants before the FGDs to establish trust. Direct communication with all participants and their parents was conducted by phone and at an informal meeting. The study objective was explained, information about the study was given, and the students were assured that their personal identity was confidential from the public, other students, and teachers. The teachers were excluded from the FGD, and the participants had the opportunity to explore their perceptions freely. Before the FGD, we informed all participants that they had equal opportunity to express their opinions. We also emphasized that every comment was useful and appreciated in developing a smoking prevention program.

Data collection was conducted in January 2019 in the three junior high schools. FGDs were conducted in these three locations (FGD A, B, and C) and each FGD involved eight students. Each FGD was conducted in the school and lasted for 60–90 min. Three cycles of FGD is considered adequate to obtain data saturation. The justification for this was also based on previous research in which FGDs were used as the data collection method. The interviews were run by the principal researcher, a senior lecturer from the Faculty of Nursing, Universitas Syiah Kuala. She has good knowledge about qualitative studies and is also experienced in running FGDs in the community. The discussion protocol was developed by the researchers, based on the reviewed literature, and was discussed in a group meeting with several experts in qualitative research before the FGDs. The FGD process was guided by the researchers with an expert-validated protocol. The interviews were audio-recorded and transcribed verbatim by two research assistants with a master’s degree and good knowledge about FGDs and qualitative studies. The FGDs were formulated to obtain detailed information about adolescents’ perceptions, opinions, and feelings about smoking. The FGD began by asking questions about perceptions on smoking: “What do you think about smoking?” and next question asked “Why do adolescents smoke a cigarette?” and they were further asked to describe the factors that predisposed the behavior. The moderator (principal researcher) delivered the questions to all participants. Then, each student was given enough time to explore their opinions and perceptions.

### Data analysis

The data were analyzed manually using inductive content analysis, with the steps carried out systematically, based on the stages of qualitative data analysis and the various parties involved [23]. The information from all FGDs was combined and considered as the unit of analysis and transcribed verbatim. The transcripts were read repeatedly by three experts in qualitative research and the researchers to obtain an overall understanding. Then, the data were broken into units of meaning and labeled with codes. The codes were compared sequentially based on similarities and differences and further formulated into sub-themes and themes, before being translated into English. Back translation was done, and two language experts assisted the researchers to ensure the best semantic equivalent and accuracy between Bahasa Indonesia and English.

### Ethical considerations

This study obtained ethical approval from the Research Ethics Committee of the Faculty of Nursing, Universitas Syiah Kuala, Banda Aceh, Indonesia. Participants had the right to refuse to be involved at any time. They knew the research objective and that their personal identity was kept confidential.

## Results

### Characteristics of the participants

Participants in this study included three focus group discussion involving 24 male students in total. Most of the participants were 13 years old; the minimum age was 12 years and the maximum age was 18 years. Most of the participants and their parents were smokers (79,17% and 83,33%)..

This study explored the perspectives of Muslim adolescent’s on smoking habits. Based on the results of the study, the adolescents’ perspectives were grouped into two themes: perception about smoking and factors related to smoking. Perception about smoking was further divided into three sub-categories: smoking is a social habit, contradictive feelings, and Islamic perspective on smoking. The second theme was based on factors related to smoking and was divided into four sub-themes: peer pressure, parents’ smoking status, curiosity, and masculinity.

### Perception of smoking

The perception of smoking was the main theme when participants discussed smoking behavior.

#### Smoking is a social habit

Smoking is perceived as a social habit in the Acehnese community, as seen from the following statement:

I think smoking is conventional. [FGD A, P1]

Other participants also confirmed that smoking is a social habit and usual behavior in the community:

… it is normal to see someone smoking cigarettes in the community. [FGD A, P2, P5, P7]

Therefore, the results illustrate that participants perceive smoking behavior as a societal practice. Hence, it was not considered a violation of any the rules and customs in the local community.

#### Contradictive feelings about smoking

Contradictive feelings are a discrepancy between behavior and perception. Therefore, the desire to stay away from cigarettes is evident from one participant’s statement. They did know about the negative effects of smoking but they were unable to say no to a cigarette from a friend. The phenomenon is known as a contradictive feeling among adolescents, where in the one hand, they want to reject cigarettes, but on the other hand, they face a situation that makes them unable to do. This was interpreted from the participant’s statement:

I know smoking is not good and it causes cancer, but I smoked a cigarette because I could not stand (unable reject) to see my friend smoking beside me. I desired to smoke when I hang out with my smoking friends, we enjoy smoking together. [FGD A, P1; FGD B, P13]

Smoking can cause cancer … but I smoked for the first time in my life out of curiosity about the taste of cigarettes. Now, I am addicted, I smoke everyday and I enjoy smoking. [FGD B, P10]

#### Islamic perspective on smoking

Based on our findings, the Islamic perspective consists of three sub-themes: forbidden (*haram*), acceptable (*halal*), and acceptable but better to avoid (*makruh*). The forbidden theme was reinforced by the following statement:

… based on my knowledge about Islam, smoking is not good because it is haram. [FGD B, P9]

This perspective is further reinforced by other respondents:

In Islamic law, someone who commits suicide is definitely going to hell and smoking also means that you damage your body and it is like you are committing suicide. [FGD B, P9]

In the view of Islam, smoking is not good because it damages our health; smoking also causes cancer. [FGD B P13, P16; FGD C, P22]

Other group opinions stated that smoking is perceived as makruh, indicating that it is acceptable but it should be stopped. This type of perspective serves as a reference for adolescents to make a choice to initiate or stop the habit, as shown by the following statements:

Smoking is makruh for Muslims because it is not prohibited in Islam, but it will be better if someone does not smoke. [FGD A, P8; FGD B, P11, P12]

As a Muslim, we can be a smoker or non-smoker, because smoking is makruh for us. [FGD A, P1, P2, P3, P4, P5, P6]

This research finding also indicated that Acehnese adolescents who portrayed this behavior were associating with the character of religious scholars as the role models for the community. This was confirmed by the following statement:

Muslims smoke because they see numerous religious scholars also smoke … [FGD C, P17, P18, P19]

This implies that the role of faith in providing exemplary behavior for an adolescent is significant because of the high tendency to imitate.

Besides haram and makruh, the Islamic perspective on smoking was interpreted based on the effects and benefits of smoking as analyzed from the following statement:

I know about Islamic laws on smoking; it depends, smoking is acceptable, but if it causes dizziness, then it is forbidden and considered as a sin. [ FGD B, P15; FGD C, P24]

This student was skeptical about the impact of smoking on health because teenagers tend to observe the short-term influence, immediately after smoking a cigarette.

This sub-theme indicates that some youths do not just follow the arguments put forward by their peers, they also elaborate on different opinions. This study indicated that some of participants agreed that the Islamic perspective on forbidding smoking depends on the immediate impact of smoking as analyzed from the following statement:

Smoking can be haram for Muslims, it can become makruh because ... if we fall sick. Hence, the law is haram, but if nothing happens to the body, then makruh law is adopted. [FGD A, P2; FGD B, P15].

#### Smoking-related factors

This study indicated that factors related to smoking for adolescents included peer pressure, parents’ smoking status, curiosity, and masculinity.

#### Smoking because of peer pressure

Peer pressure is defined as a condition whereby friends persuade or influence an adolescent into partaking in a habit. However, due to the strong ties and the tendency to behave in a similar manner as their acquaintances, adolescents obtain recognition and are thus considered a part of the group. This sub-theme was reinforced by the perception that avoiding smoking makes them a ridicule to their peers as illustrated by the following statement:

If I don't smoke, I feel ridiculed by friends in my peer group. [FGD C, P21]

Then, another participant stated:

The first time, I smoked because at that time I sat with my friends, and all of them were smoking cigarettes; so I did too. [FGD B, P13]

These results further indicate that teenagers adopt this behavior because of peer and group pressure in order to survive in their units.

#### Smoking because of parents’ smoking status

Teenagers with parents who smoke tend to believe that smoking is acceptable because their parents have been role models for decision making. Acehnese teenagers tend to adopt the behaviors conducted by their guardians, as analyzed from the following statement:

I smoke because my father smokes in front of me. Every day I see my father smoke at home, I think smoking is enjoyable and acceptable. [FGD B, P10]

Another participant also stated

If parents smoke cigarettes in front of their children, it seems like smoking is acceptable and not forbidden at all; so the children will also smoke. [FGD A, P7]

#### Smoking is a symbol of masculinity

Masculinity can be defined as qualities or attributes regarded as characteristic of men, such as being handsome and muscled, and likely applies in many countries; adolescents in Aceh comprehend the concept of masculinity as an important issue. This study identified that smoking was affiliated with masculinity, and teenagers perceived that a smoker showed specific characteristics such as strength, courage, and independence. This is inferred from the following statement:

If you don't smoke, you do not look like a real man, and you are not mature. Hence, you are ridiculed by friends to be weak man. [FGD C, P21, P22]

This perception predisposes the adolescent to greater risks, and they maintain their smoking habit to portray themselves as strong men. The stigma of being a weak man for a non-smoker is confirmed from the following statement:

When I did not smoke, one of my friends asked me, why don’t you smoke, you look very weak?…Smoking help us to be a strong man. [FGD C, P22]

Therefore, smoking was perceived to be a way for the adolescent to be recognized by their peers as having masculine characteristics.

#### Smoking out of curiosity

Adolescence is a transition period, characterized by strong curiosity about everything, including trying cigarettes, as found in the teenagers in Aceh. The phenomenon was confirmed from the following statement:

The first time I smoked was because I was curious about the taste of the cigarette. I tried it, which got me addicted. [FGD B, P10]

Then, this sub-theme was strengthened by another participant:

I smoked a cigarette because of its good smell, which stimulated me to take it. [FGD A, P3, P7]

This result reflects a connection between curiosity and the smoking habit among adolescents in Indonesia.

## Discussion

In this qualitative research, teenagers expressed their perceptions of smoking, based on social contexts and Islamic perspectives. The results are important for educators and health practitioners, especially those involved in smoking prevention efforts and health promotion, to assist in the development of effective intervention programs appropriate for participants’ religion. The following discussion is presented according to the sub-themes.

The outcomes of this research indicate that smoking is perceived as a social habit that exists in the local community. Adolescents consider it to be acceptable and not contrary to the norms of society. These results verify previous studies, carried out in Mexico, where smoking was generally described as socially acceptable for men [5]. This finding is also consistent with a previous study, which reported that when smoking has been accepted in social networks, especially among family members and friends, prevention is likely to be ineffective [25].

Muslim teenagers in Indonesia apparently have a similar perception about smoking as adolescents in other countries. They tend to smoke because it is considered an acceptable habit in their social environment. This is in line with previous research showing that teenagers were sensitive to friends who smoked, and also within a wider network of friends [26]. Other investigations have reported that young adults have a special relationship with smoking. They believe that smoking can enhance their social relationships [27].

The results also showed that teenagers experience contradictive feelings in relation to smoking, which are linked to the information they have about its consequences. However, warning labels can reduce smoking behavior in the short term. Hence, it is important to disseminate information to the public about the effects of smoking on health [28]; although teenagers are a vulnerable age group, easily influenced by various positive and negative issues, some partake in this habit because there is no direct immediate impact on health [28]. Furthermore, warnings about the dangers attributed to smoking cause contradictive feelings, as they incite fear. Therefore, contradictory warning labels are more meaningful if targeted at current smokers and can also prevent youth from starting the behavior [28]. However, this is an important issue to consider when initiating a program. A previous study illustrated the importance of negative affect indirectly motivating the desire to stop smoking, hence, serving as a source of information to influence adult smokers and non-smoking adolescents to accept the health risks [29].

Contradictory feelings also arise because of the inability of adolescents to control their behavior according to their desire. Smoking is a voluntary response to unintentional desires, which in some cases, involves neglecting the use of voluntary efforts to resist impulsive actions [30]. The results of this study indicate the experience of contradictive feelings related to smoking. Sometime, they still feel reluctant to be or not to be a smoker. This theme also relates to differences in views about the laws governing the behavior among scholars.

The results of this study showed that some participants perceived that smoking is forbidden according to Islamic law. The major reason why smoking is prohibited based on Islamic perspectives is that it has adverse effects on health and can lead to death.. However, some teenagers in this study viewed smoking as makruh, which means it is allowed, although it is better to not smoke. Furthermore, this result agrees with a previous study, which stated its permissibility, whereby Muslim smokers perceive the behavior as acceptable in Islam – hence, Makruh – although, if it is too much, it could be haram [31]. This awareness fuels the desire to smoke and the desire to continue to do so among teenagers, eventually creating dependence.

Adolescents tend to observe the short-term impact of smoking and therefore adopt the behavior because there is no immediate effect on health, indicating the inadequacy of the information available to them about the dangers. Furthermore, this study is in line with previous studies, which reported that a number of secondary school students in urban areas lack specific knowledge about smoking-related diseases [31], and other studies that showed the main motivation is to be social [32]. The dangers of tobacco are seen to be low, and an independent risk factor is a perception that friends and other students take substances and drink alcohol [33]. Based on the results, Muslim adolescents who consider this habit as makruh and not forbidden are more at risk of participating. Hence, preventive efforts are urgently needed to curb smoking initiation, which leads to regular tobacco use and dependence.

This finding is also in agreement with previous studies on the factors related to smoking, where the influence of friends was observed as an important factor. The main related factors include having friends who are addicted, being offered cigarettes, and ease of access [34]. Moreover, this study found that peer pressure is also a significant issue related to smoking among adolescents. This agrees with previous studies that demonstrated the importance of peer pressure on this habit among students [35]. Adolescents feel more comfortable sharing similarity with their friends, including smoking behavior; those who have such friends tend to behave likewise [36].

Parenting seems to be an important stimulus for adolescents in the period of conduct development [36]. As seen generally, Muslim teenagers in Indonesia are strongly influenced by guardians, because they serve as role models as shown in this study. This agrees with previous studies, where families play a strong role and therefore affect adolescent smoking behavior [37].

Based on the result of this study, masculinity is a significant issue related to smoking. Participants were determined to smoke because it made them appear strong and mature. This is supported by previous research, which found that the desire to be manlike was associated with initiation of smoking [38]. However, another study from Canada perceived smoking as unhealthy and uncool [17]. Another factor contributing to smoking based on this study is curiosity. This finding is in line with a previous study, which found that smoking was driven by curiosity [39].

Our study adds new information about the adolescents’ perspectives on smoking, which included social, health and religion aspects. The findings of the study open up new avenues for further qualitative research related to smoking behavior among adolescents. However, this study may have a limitation related to the participants’ characteristics. Most of the participants in this study were smokers. It is possible that opinions from smokers dominated the discussions and influenced the research findings.

## Conclusion

The results of this study illustrate that adolescents consider smoking as a social habit, a contradictive feeling, and from the Islamic perspective, it is deliberated by most teenagers to be makruh. The initiators were peer pressure, parents’ smoking status, curiosity, and masculinity. We recommend that health professionals who are interested in developing smoking prevention programs should consider adolescents’ perspectives on the smoking habit in relation to health, social and religion issues that could increase the effectiveness of a program.

## Data Availability

The datasets used in the current study are available from the corresponding author on reasonable request.
